# Altmetric Analysis of Artificial Intelligence Articles in Plastic Surgery

**DOI:** 10.1055/a-2223-5458

**Published:** 2024-01-29

**Authors:** Brennan Bogdanovich, Pearl Shah, Parth A. Patel, Tommy Bui, Carter J. Boyd

**Affiliations:** 1 Medical College of Georgia, Augusta University, Augusta, Georgia; 2 Hansjörg Wyss Department of Plastic Surgery, NYU Langone Health, New York, New York

Artificial intelligence (AI) is becoming increasingly relevant and integrated into the medical space. Current adoption in AI is distinct from prior attempts, as computer processing power, larger data storage libraries, and current AI workforce talent outweigh previous capabilities. These advances have enabled AI-based systems to flourish within health care. In a report produced by Accenture, it is estimated that AI, by 2026, has the potential to save the health care industry over $150 billion annually. 1 Plastic surgery specifically could leverage AI to optimize patient care.

Given this rapid expansion of AI in the literature, it is necessary to identify the most salient articles in the field. Traditionally, bibliometric analyses have enabled identification of seminal articles through quantification of citation count. However, citation count, while useful, fails to account for social dissemination. We provide an alternative perspective from traditional bibliographic analysis by using Altmetric Attention Scores (AAS) to determine the online social influence of AI-related plastic surgery articles. Intended to be complementary to traditional, citation-based metrics, AAS reflects the digital attention a research article is garnering across multiple sources, including, but not limited to Twitter, news outlets, Facebook, Google + , LinkedIn, Reddit, etc. 2

The Web of Science database was searched with a combination of Boolean operators and descriptive terms to identify articles relevant to AI and plastic surgery. Articles were manually examined to ensure relevance to the present analysis. In total, 285 articles were identified from the database search and 266 were eligible for screening after removing duplicates. After eliminating irrelevant articles, 141 articles remained. AAS, which measures the social dissemination of an article, was determined using Altmetric Explorer. Articles were ranked by citation count and AAS, and their characteristics were analyzed using the Pearson correlation coefficient, Mann–Whitney U test, Kruskal–Wallis test, and Fisher's exact test, where appropriate. *p* < 0.05 was considered statistically significant.

The mean AAS of the 50 most disseminated articles online was 11.3 ± 19.2, primarily driven by mentions on Twitter (12.2 ± 16.5). No correlation was identified between AAS and citation count (r = 0.13; *p* = 0.38). No articles were published prior to 2014, with 68% published between 2020 and 2022 ( Supplementary Table S1 , available in the online version only). Forty-two percent of articles were open access, a similar proportion relative to the 50 most cited articles (44%; *p* > 0.99). *Plastic and Reconstructive Surgery* was the most common journal of publication for the 50 most disseminated articles online (26%) and articles published in this journal accrued greater AAS relative to other journals ( *p* = 0.04). A majority of articles (64%) were multi-institutional collaborations and 34% were multinational collaborations ( Supplementary Table S2 , available in the online version only). The most common subspecialty of social interest was general/burn (28%; Supplementary Table S3 , available in the online version only). First authors were predominantly male (84%) and from the United States (54%).

As noted by Elmore in 2019, AAS presents with certain limitations, including but not limited to an absence in the ability to analyze the quality of an article and difficulty with field normalization. Additionally, the Altmetric algorithm uses sites like Facebook, Reddit, and Twitter, but does not use TikTok or Instagram. It is also important to note that AAS is not related to the scientific importance of an article but rather the social influence. However, analyzing the social influence acts as an important complementary measure to traditional, citation-based metrics. 2

The collaborative effort of plastic surgeons in AI-related research was a notable finding of our analysis. Most of the plastic surgery articles analyzed were multi-institutional and greater than one-third were multinational. Moreover, while female authorship percentages in plastic surgery have been increasing according to Silvestre et al, 3 only 16% of articles published in the plastic surgery/AI space cross-section were female. Interestingly, 22% of employees in the AI workspace are female, closely mirroring our findings and pointing to a potential coexisting male bias in the field of AI. 4

There is limited literature available on this topic. Our analysis of plastic surgery-related AI papers revealed 266 articles within the Web of Science database, with 141 articles ultimately analyzed. Conversely, in a recent paper that analyzed AAS of AI in the ophthalmology literature, the authors identified 2,927 total articles. 5 Although plastic surgery is a field that prides itself on innovation, approximately one-tenth the number of articles regarding AI in plastic surgery was initially identified relative to ophthalmology. We hypothesize that the lack of available literature is due to the novelty of the subject and its potentially unclear role within the field of plastic surgery. As such, lest plastic surgeons fall behind, it is paramount for the field to discover novel means of integrating and leveraging AI within the specialty, either in clinical or business operations.
