# Supplementary material for: Altmetric Analysis of Artificial Intelligence Articles in Plastic Surgery
Source: Arch Plast Surg. 2024 Jan 29;51(2):262–4. doi: 10.1055/a-2223-5458 (PMC11001444; doi:10.1055/a-2223-5458)
Supplement: Supplementary file 1 — Supplementary Material [file 10-1055-a-2223-5458-s23may0328let.pdf]

Supplementary Material

Supplementary Table S1 Top 50 articles examining artificial intelligence in plastic surgery by Altmetric Attention Score

Number	Article title	Journal	Publication year	AAS
1	Turning back the clock: artificial intelligence recognition of age reduction after face-lift surgery correlates with patient satisfaction	Plastic and Reconstructive Surgery	2021	91
2	Making the subjective objective: machine learning and rhinoplasty	Aesthetic Surgery Journal	2020	90
3	Big data and machine learning in plastic surgery: a new frontier in surgical innovation	Plastic and Reconstructive Surgery	2016	48
4	Present and future of machine learning in breast surgery: systematic review	British Journal of Surgery	2022	44
5	A machine learning framework for automated diagnosis and computer-assisted planning in plastic and reconstructive surgery	Scientific Reports	2019	34
6	Risk of developing breast reconstruction complications: a machine-learning nomogram for individualized risk estimation with and without postmastectomy radiation therapy	Plastic and Reconstructive Surgery	2022	22
7	Facial recognition technology: a primer for plastic surgeons	Plastic and Reconstructive Surgery	2019	18
8	Facial recognition neural networks confirm success of facial feminization surgery	Plastic and Reconstructive Surgery	2020	16
9	Photographic and video deepfakes have arrived: how machine learning may influence plastic surgery	Plastic and Reconstructive Surgery	2020	15
10	A machine learning approach for automated facial measurements in facial palsy	JAMA Facial Plastic Surgery	2018	15
11	The use of emotional artificial intelligence in plastic surgery	Plastic and Reconstructive Surgery	2019	14
12	In the eye of the beholder: changes in perceived emotion expression after smile reanimation	Plastic and Reconstructive Surgery	2019	13
13	Artificial intelligent virtual assistant for plastic surgery patient's frequently asked questions: a pilot study	Annals of Plastic Surgery	2020	12
14	Machine learning applied to registry data: development of a patient-specific prediction model for blood transfusion requirements during craniofacial surgery using the pediatric craniofacial perioperative registry dataset	Anesthesia and analgesia	2021	10
15	The applications of machine learning in plastic and reconstructive surgery: protocol of a systematic review	Systematic Reviews	2020	8
16	Development of a novel scar screening system with machine learning	Plastic and Reconstructive Surgery	2022	7
17	A role for artificial intelligence in the classification of craniofacial anomalies	Journal of Craniofacial Surgery	2021	7
18	Improving face recognition performance using TeCS2 dictionary	Pattern Recognition Letters	2021	6
19	A machine learning algorithm to estimate the probability of a true scaphoid fracture after wrist trauma	Journal of Hand Surgery - American Edition	2022	6
20	Development and validation of a deep learning model using convolutional neural networks to identify scaphoid fractures in radiographs	JAMA Network Open	2021	6
21	A machine learning-based model for breast volume prediction using preoperative anthropometric measurements	Aesthetic Plastic Surgery	2022	5
22	Three-dimensional stereophotogrammetry in the evaluation of craniosynostosis: current and potential use cases	Journal of Craniofacial Surgery	2021	5

(*Continued*)

 © 2024. The Author(s).

Supplementary Table S1 (*Continued*)

Number	Article title	Journal	Publication year	AAS
23	Artificial Intelligence in plastic surgery: What is it? Where are we now? What is on the horizon?	Annals of the Royal College of Surgeons of England	2020	5
24	Personal computer-based cephalometric landmark detection with deep learning, using cephalograms on the internet	Journal of Craniofacial Surgery	2019	4
25	Personalized quantification of facial normality: a machine learning approach	Scientific Reports	2020	4
26	Frontal bone is thicker in women and frontal sinus is larger in men: a morphometric analysis	Journal of Craniofacial Surgery	2021	4
27	Use of convolutional neural networks to evaluate auricular reconstruction outcomes for microtia	The Laryngoscope	2022	4
28	Machine learning to predict individual patient-reported outcomes at 2-year follow-up for women undergoing cancer-related mastectomy and breast reconstruction (INSPiRED-001)	The Breast	2021	4
29	Improving efficiency of patient-reported outcome collection: application of computerized adaptive testing to DASH and QuickDASH Outcome Scores	Journal of Hand Surgery - American Edition	2021	4
30	Toward an automatic system for computer-aided assessment in facial palsy	Facial Plastic Surgery and Aesthetic Medicine	2020	4
31	The cluster assessment of facial attractiveness using fuzzy neural network classifier based on 3D Moire features	Pattern Recognition	2014	3
32	Artificial intelligence and machine learning in the identification of authentic and fake data presentation	Journal of Craniofacial Surgery	2019	3
33	Artificial intelligence will empower clinical judgment and common sense, not impair or replace them	Journal of Craniofacial Surgery	2019	3
34	Reply: Using artificial intelligence to measure facial expression following facial reanimation surgery	Plastic and Reconstructive Surgery	2022	3
35	Clinician and automated assessments of facial function following eyelid weight placement	JAMA Facial Plastic Surgery	2019	3
36	Artificial intelligence confirming treatment success: the role of gender- and age-specific scales in performance evaluation	Plastic and Reconstructive Surgery	2022	2
37	Cancer-on-a-chip and artificial intelligence: tomorrow's cancer management	Journal of Craniofacial Surgery	2018	2
38	Sagittal jaw relationship of different types of cleft and non-cleft individuals	Frontiers in Pediatrics	2021	2
39	Evaluation of sella turcica bridging and morphology in different types of cleft patients	Frontiers in Cell and Developmental Biology	2020	2
40	Evaluation of an artificial intelligence system for diagnosing scaphoid fracture on direct radiography	European Journal of Trauma and Emergency Surgery	2022	2
41	Impact of artificial intelligence in the brave new medical world: no time to go back	Journal of Craniofacial Surgery	2018	2
42	Facelift surgery turns back the clock: artificial intelligence and patient satisfaction quantitate value of procedure type and specific techniques	Aesthetic Surgery Journal	2021	2
43	3D photography to quantify the severity of metopic craniosynostosis	The Cleft Palate - Craniofacial Journal	2022	2
44	Machine learning models for genetic risk assessment of infants with non-syndromic orofacial cleft	Genomics Proteomics and Bioinformatics	2018	1
45	Assessment and prediction of response to neoadjuvant chemotherapy in breast cancer: a comparison of imaging modalities and future perspectives	Cancers	2021	1

Supplementary Table S1 (*Continued*)

Number	Article title	Journal	Publication year	AAS
46	A generative adversarial network approach to predicting postoperative appearance after orbital decompression surgery for thyroid eye disease	Computers in Biology and Medicine	2020	1
47	Gene-gene interaction among WNT genes for oral cleft in Trios	Genetic Epidemiology	2015	1
48	Quantifying the severity of metopic craniosynostosis: a pilot study application of machine learning in craniofacial surgery	Journal of Craniofacial Surgery	2020	1
49	Quantification of head shape from three-dimensional photography for presurgical and postsurgical evaluation of craniosynostosis	Plastic and Reconstructive Surgery	2019	1
50	The Auto-eFACE: machine learning-enhanced program yields automated facial palsy assessment tool	Plastic and Reconstructive Surgery	2021	1

Abbreviation: AAS, Altmetric Attention Score.

Supplementary Table S2 Region and country of origin of senior authors of top 50 articles by Altmetric Attention Score

	Proportion of articles in top 50	Mean AAS ± SD
North America		14.31 ± 23.20
United States	54%	
Canada	4%	
Asia		3.42 ± 1.93
Japan	4%	
Turkey	4%	
China	2%	
India	2%	
Iran	2%	
Pakistan	2%	
Qatar	2%	
Republic of Korea	2%	
Saudi Arabia	2%	
Taiwan	2%	
Europe		12.63 ± 16.65
United Kingdom	8%	
Estonia	2%	
Finland	2%	
Italy	2%	
Netherlands	2%	

Abbreviations: AAS, Altmetric Attention Score; SD, standard deviation.

Supplementary Table S3 Subspecialty distribution of top 50 articles by Altmetric Attention Score

	Proportion of articles in top 50	Mean AAS ± SD
General/Burn	28%	12.43 ± 13.45
Craniofacial	22%	3.36 ± 2.91
Breast	14%	11.57 ± 16.03
Aesthetic	12%	32.83 ± 44.70
Microsurgery	12%	6.50 ± 5.93
Hand	8%	4.5 ± 1.92
Gender-affirming	2%	16 ± NA
Ophthalmic	2%	1 ± NA

Abbreviations: AAS, Altmetric Attention Score; SD, standard deviation; NA, not applicable.

 © 2024. The Author(s).
